# The Remarkable Increase in the Invasive Autumn Fern, *Dryopteris erythrosora*, One of the World’s Most Marketed Ferns, in Eastern North America

**DOI:** 10.3390/plants14152369

**Published:** 2025-08-01

**Authors:** Robert W. Pemberton, Eduardo Escalona

**Affiliations:** 1 Independent Researcher, 2275 1st Ave. NE, Atlanta, GA 30317, USA; 2 Independent Researcher, E 6323 S Rosebury Ave 1 W, Saint Louis, MO 63105, USA; eem1958@gmail.com

**Keywords:** citizen science, displacement, escaped ornamental, forests, herbarium specimens, iNaturalist, natural areas

## Abstract

Autumn fern, *Dryopteris erythrosora*, is the most marketed temperate fern in the world. The rapid increase and spread of this recently naturalized fern in North America was determined and mapped using 76 herbarium specimen records and 2553 Research Grade iNaturalist posts. In 2008, it was recorded in two states, but by 2025, it was found in 25 states in the eastern United States and Ontario, Canada. At the end of 2017, there had been only 23 iNaturalist posts, but this grew to 511 by the end of 2020 and 2553 by May 2025. The great increase in the number of iNaturalist posts is thought to be due to the real geographic spread and an actual increase in the abundance of the fern, as well as recognition of the fern by iNaturalists, and the increase in the number of iNaturalists. The spread and great increase are probably related to the high level of marketing, which introduces plants to the environment, and to biological characteristics of the fern, including apogamy and polyploidy, and possibly natural enemy release, which allows it to flourish in new environments and to displace native plants. This novel study demonstrated citizen science’s (iNaturalist’s) great value in detecting the naturalization and spread of alien plants.

## 1. Introduction

Marketing of alien plants is widely recognized as the primary pathway for invasive plant introduction [1]. The length of time non-native horticultural plants have been marketed is related to the degree of naturalization and invasiveness of plants [2]. One of the most ubiquitous threats to biodiversity today is the conversion of native plant communities into plant assemblages dominated by non-native species [3].

McColloch-Jones et al. [4,5] reported that *Dryopteris erythrosora* (D.C. Eaton) Kuntze, known as autumn fern in English, is the most marketed of temperate zone ferns. Due to this high-level market intensity and its recent naturalization in some countries, these authors described it as one of four ferns of particular concern for its potential to spread further and invade. The first herbarium specimen collected in Georgia of autumn fern was in 2012 [6]. In 2021, Pemberton [7] found autumn fern at 56/58 surveyed natural preserves and parks in the Atlanta/Gainesville region of Georgia. The fern was abundant at 28 of the 56 of these sites, often dominating ground level and creekside vegetation.

**Figure 1:**
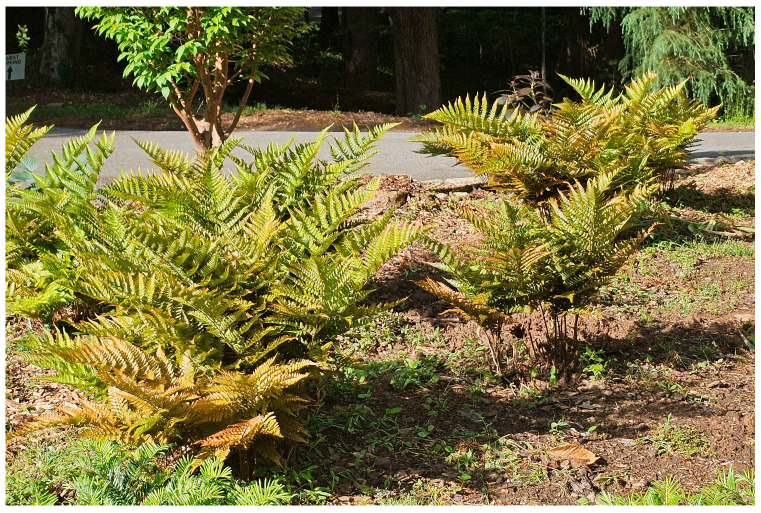
Garden planting of autumn ferns, *Dryopteris erythrosora*, showing their appealing upright growth and the coppery-orange coloration of the new leaves.

Autumn fern is native to Japan, China, South Korea, and Taiwan [8]. It is extensively planted in residential gardens, in the outdoor spaces of businesses and public parks, and in other settings. It is an appealing horticultural subject because it is fairly large, growing to 1 m tall, is evergreen, has attractive orange- or copper-colored new growth, is pest-free, and requires little to no maintenance or irrigation [9] (Figure 1).

The fern’s astonishingly rapid spread and ability to dominate natural environments in the Atlanta region suggest that it has the potential to naturalize and spread elsewhere in North America. The goal of this study was to define the fern’s known naturalization and spread in North America. Autumn fern herbarium specimen locations and collection dates were used with iNaturalist citizen scientists’ posts of photographs of the fern, with dates and GPS locality data, to map the occurrence of the fern in seven different time periods. From the first detection of escaped autumn fern plants in Arkansas in 2007 [10], it spread to 11 states by 2017, and to 25 states and Ontario in Canada by May 2025. By the end of 2017, there were only 63 iNaturalist posts of the observer photographs of fern, which grew to 2553 posts by early May 2025, in less than eight years. This rapid and enormous increase in the occurrence and the geographic spread of autumn fern is thought to be due to the actual increase in autumn fern, as well as recognition of the fern by iNaturalists, and an increase in the number of iNaturalists. This study demonstrated citizen science’s (iNaturalist’s) great value in detecting the naturalization and spread of alien plants.

The actual increase in the fern is probably related to the high level of marketing it experiences. This great increase and spread of the fern, which is largely unrecognized, has unknown ecological significance, but its dominance in some areas suggests it is displacing native plants. Autumn fern is most likely displacing native vegetation and probably the native biological associates (insects, etc.) of these plants. Research needs to continue to monitor the fern’s rapid increase and spread. Future research should address autumn fern’s ecological impacts, possible control methods, and how best to create public awareness of this popular fern’s negative aspects.

## 2. Results

**Figure 2:**
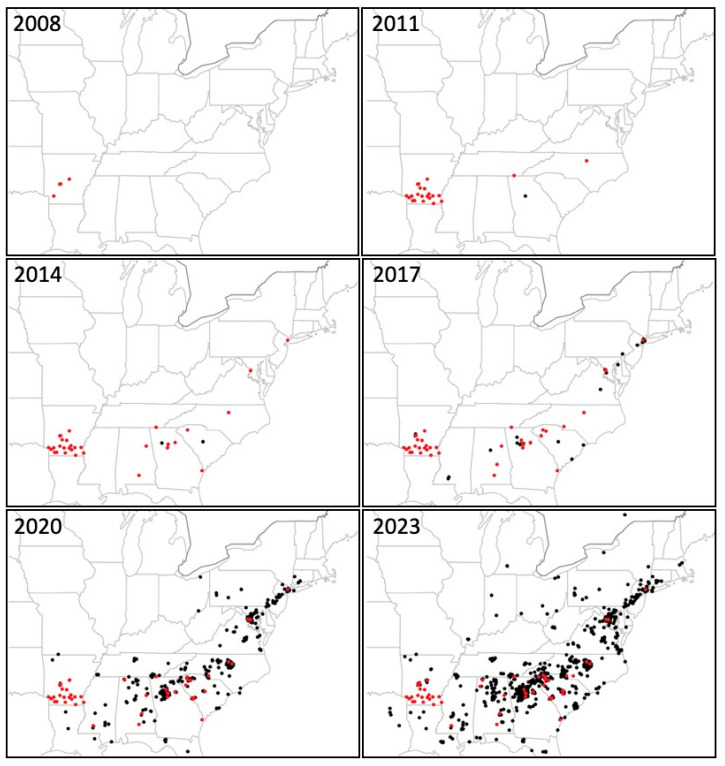
Distribution and spread of autumn fern, *Dryopteris erythrosora,* at three-year intervals ending on 31 December of the year indicated, except for the 2023 period, which ended on 1 October 2023. Red spots are herbarium specimen collection locations. Black spots are iNaturalist post locations.

**Figure 3:**
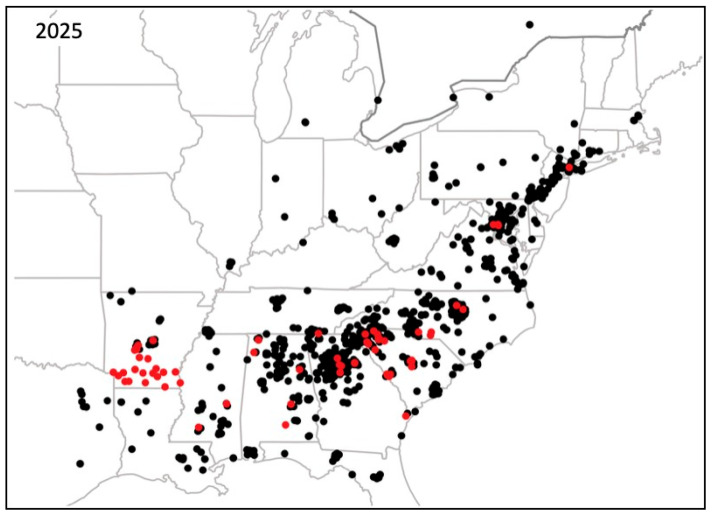
Distribution of autumn fern, *Dryopteris erythrosora*, as of 8 May 2025. Red spots are herbarium specimen collection locations. Black spots are iNaturalist post locations.

Figure 2 shows six maps of the autumn fern’s distribution from 2008 through 2023. In 2008, only two states, Arkansas and South Carolina, were known to have the fern based on herbarium collection records. In 2011, four states (Arkansas, Georgia, North Carolina, and South Carolina) had the fern based on herbarium records and the first iNaturalist post in Georgia in 2010. In 2014, the number of states with the fern doubled again to eight, based on herbarium records and five iNaturalist posts. By this date, the fern occurred beyond the southeastern United States for the first time, having been found in New York. In 2017, the fern was found in 11 states, spreading to more states along the east coast, including Virginia, Maryland, Pennsylvania, and New Jersey. In 2020, the fern occurred in 18 states, spreading farther northeast to Delaware and Connecticut, west to Ohio in the Midwest, and more widely in the South to Florida, Louisiana, and Texas. In 2023, the fern was found in 23 states, reaching farther northeast to Massachusetts, and to more midwestern states including Indiana, Illinois, and Michigan. During this period, the first iNaturalist post was made for the fern in Canada. This was in Ottawa, Ontario, on 6 June 2022. By May of 2025, autumn fern was found in 25 states (Figure 3), occurring in Kentucky and West Virginia, as well as an additional two sites in southern Ontario, Canada. In just 18 years, the number of states with naturalized autumn fern plants increased from 2 to 25, and it also spread to Canada.

The number of occurrences of the fern also increased dramatically with time, and the type of records used also changed. iNaturalist was created in 2007 and had its first post of any type in 2008 [11]. The first post of autumn fern was in Georgia in 2010, which occurred two years earlier than the first herbarium specimen was collected in 2012. Prior to and through 2017, the majority of the occurrences of autumn fern mapped were based on herbarium records. There were only five iNaturalist posts for the fern by 2014. The cumulative number of iNaturalist posts rose to 23 by the end of 2017. Then the number of posts increased dramatically to 511 by the end of 2020, rocketing to 1473 posts by October 2023, and 2553 posts by May 2025. The fern’s geographic expansion reached 23 of the 25 states it occurs in 2025 by 2017, but the abundance of the fern, based on the number of iNaturalist posts for the period [11], was low. The fern’s abundance began to increase strongly during the 2020 period, when there were 488 new posts for the three-year period ending in 2020. The increase in abundance can be shown by calculating the number of new iNaturalist posts per month for each period. In the period ending at the end of 2017, there were 0.70 posts per month. By the end of 2020, there were 14.8 posts per month, which doubled to 29 posts per month by October 2023, which doubled again to 55 posts per month by May 2025. In contrast, the collection of herbarium specimens did not show a continuous or large increase with time, so the number of herbarium specimens does not appear to reflect the abundance of the fern. Peck and colleagues collected 24 specimens of autumn fern in Arkansas from 2007 through 2011. Following that, there were from two to six specimens collected each year in the United States, mostly in the Southeast, through 2023.

The statistical analysis used to complement the descriptive analysis, a Poisson regression model to the iNaturalist post counts across five time periods (2012–mid-2025), adjusting for the number of months in each interval, produced the following results: coefficient for mid_year, 0.3636; SE, 0.008; z-value, 45.54; and *p*-value, <0.0001. This corresponds to an estimated 43.8% increase in the expected number of iNaturalist posts per month for each additional year (*exp*(0.3636) = 1.438). These results provide quantitative confirmation of the striking increase in autumn fern’s abundance suggested by the raw counts and maps.

## 3. Discussion

### 3.1. Study Methods

This large increase in iNaturalist posts is probably due to a combination of factors. First, the actual increase in the occurrence and abundance of the fern, then its recognition by those posting on iNaturalist, followed by the increase in the number of iNaturalists. As noted above, the number of iNaturalist posts doubled each month from January 2018 through May 2023, while the number of iNaturalist observers is calculated to be an increase of ca. 50% per month during the same time period, indicating an actual increase in the number of ferns. Unlike many ferns, the autumn fern is easy for naturalists and other non-specialists to recognize because of the unique orange-copper coloration of its new growth. The new growth is most prominent during the spring flush, but new fronds with this distinctive coloration continue to be produced throughout the growing season. Although large numbers of iNaturalist posts of autumn fern exist, it has been reported that most iNaturalist observers are typically focused on a particular group, especially insects and plants, and rarely make observations of the same species twice [12]. This phenomenon probably limited the number of posts of autumn fern from being made, suggesting that the occurrences of the fern are underreported. It is unknown whether the number of botanists increased during this time, but the collection of herbarium specimens seems to have been idiosyncratic. It was surprising that only one of the five largest herbaria, the US Natural History Museum, held specimens of the fern, and it had only four specimens collected in North America. The use of herbarium specimens to look at the spread of invasive plants is biased by differing intensities of collections [13]. For instance, the numerous specimens collected in Arkansas, which we mapped, reflect the activity of pteridologist James Peck [14] and his colleagues as well as the occurrence of the plants. The apparent recency of autumn fern’s naturalization and spread is probably why so few herbarium specimens have been collected. The 2553 iNaturalist posts provided 30 times more locality and date data than the 76 herbarium specimens did. The iNaturalist online citizen science initiative was particularly valuable in detecting the naturalization and spread of autumn fern, and probably other plants that can be easily identified from photographs.

The Poisson regression approach was well suited to the structure of the data and the observed exponential growth pattern. While it confirmed a highly significant exponential trend, the actual observed increase was even steeper. The monthly rate of iNaturalist posts rose from just 0.17 in the 2012–2014 period to nearly 55 by mid-2025. The largest jump occurred between 2015–2017 and 2018–2020, with a more than twentyfold increase in monthly posts. These bursts of growth suggest that the spread of *Dryopteris erythrosora* may be accelerating beyond what a smooth model predicts, likely due to a combination of real expansion, increased awareness, and citizen science participation.

### 3.2. Prior Collections of Naturalized Autumn Fern

The first collections of autumn fern in North America were in Arkansas in 2007 by J. Peck (07-2041 in BRIT) and D. Crank (07-124 in BRIT) [10], and collections continued in subsequent years at many other localities in Arkansas [14]. Other early collections were made in Tennessee in 2009 by G. Morton (UCHT006063), North Carolina in 2010 (Rothfels 3959 in DUKE), in Virginia in 2011 (Simmons 3391 in AVCH), Alabama in 2012 (Spaulding 13,394 in NCU), and Georgia by J. Allison in 2012 (FLAS275780). Autumn fern has been recently collected in five counties in the Atlanta area, and in two counties in and near Athens, Georgia [6]. Umstead and Diggs [15] found large numbers of autumn ferns growing in a woodlot in Alpharetta in the northern suburbs of Atlanta in 2017. *Dryopteris erythrosora* is naturalizing in suburban woods and ravines in the Southeast and seems to be on its way to becoming an aggressive invasive [6,16].

### 3.3. Prior Research on Autumn Fern’s Naturalization

**Figure 4:**
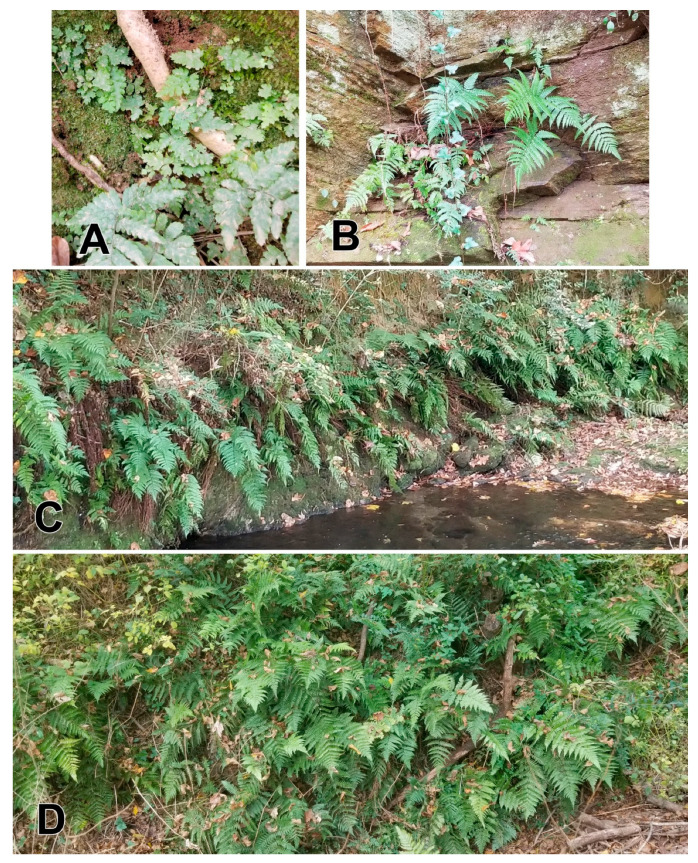
Naturalized autumn fern, *Dryopteris erythrosora.* (**A**) Sporelings on a clay creekbank in an Atlanta, Georgia, nature preserve. (**B**) Plants growing on a cliff face in a Gainesville, Georgia, park. (**C**) Plants lining a creek bank in a Decatur, Georgia, park. (**D**) Plants covering a mesic forest hillside in an Atlanta nature preserve.

McColloch-Jones et al. [4,5] reported that *Dryopteris erythrosora* (D.C. Eaton) Kuntze, known as autumn fern in English, is the most marketed of temperate zone ferns as defined by the number of commercial nurseries selling the fern. Due to this high-level market intensity and its recent naturalization in some countries, these authors described it as one of four ferns of particular concern for its potential to spread further and invade. In 2021, Pemberton conducted surveys for plants of McColloch-Jones et al.’s [4,5] four ferns of most concern as potential invaders in the Atlanta/Gainesville region in northern Georgia [7]. In addition to autumn fern, these are Japanese holly fern, *Cyrtomium falcatum* (L.f.) K. Presl, blackwood fern, *Dryopteris cycadina* (Franch. & Sav.) C. Chr., and Japanese or Korean tassel fern *Polystichum polyblepharon* (Roem. ex Kunze) C. Presl. Fifty-eight forest sites in nature preserves and natural situations in parks in eight different counties were surveyed for these ferns. Autumn fern was found at 56 of the 58 sites in seven of the eight counties surveyed. A few months after the survey, autumn fern was found at the 57th site in the eighth county. At half of the sites (28/57) where the fern was found, it was abundant. Autumn fern was also often the most abundant fern at many of these sites and dominated some environments (Figure 4). Many sporelings of the fern commonly grow from gametophytes on clay banks of creeks (Figure 4A). Autumn fern can grow on nearly vertical rock walls (Figure 4B). Infestations of the fern frequently occur along creeks (Figure 4C) and on hillsides in mesic hardwood forests (Figure 4D), where the plants proliferate to the degree that other vegetation is often excluded.

### 3.4. Market Influence on the Naturalization

Although the fern’s increasing distribution is described as spread, the occurrence of this fern in an increasing number of states probably does not represent actual spread from the Southeast, where it first naturalized. This is because the intense marketing of the fern throughout eastern North America continually created plantings of new fertile plants that could promote local naturalization and spread. Given the widespread cultivation of autumn fern for many years [9,17], it probably was naturalized in other states but not recognized. The market intensity of autumn fern has been and is high. The Plant Locator Western Region [18] listed 31 specialty fern nurseries selling autumn fern in 2004. An online Google search of outlets selling the fern in November of 2023 found more than 70. These included both in-store and online markets. Visits to the biggest brick-and-mortar stores such as Walmart, Lowe’s, and Home Depot in the Atlanta area found large numbers of autumn fern plants for sale. Attempts to obtain sales information on autumn fern from important national growers of temperate fern, such as Costa Farms and Monrovia, as well as Pikes Nurseries, a prominent chain of Atlanta regional nurseries, were not successful.

### 3.5. Biological Influences on the Naturalization

In addition to the intense marketing that introduces large numbers of ferns to the environment, other factors probably influence the naturalization of autumn fern. *Dryopteris erythrosora* is autogamous [19], which means that it does not have a sexual stage. A single spore can develop into a plant, and this plant, into a population, even in environments without free water. Apogamy also results in more rapid growth [9]. The spores of autumn fern are viable for one year [20]. It is a polyploid [21], a feature that appears to be related to greater naturalization of plants [22]. The abundance and rapid spread of *D. erythrosora* may be due in part to the fern’s escape from its natural enemies: the “enemy release hypothesis” [23]. Within its native distribution of Japan, the fern is heavily browsed by stathmopodid moths (micro-Lepidoptera), which do not occur in North America. Sawamura, Kawakita, and Kato [24] found that up to 50 larvae of these moths were found on a single leaf, and 70% of the sporangia were commonly destroyed by the feeding of the larvae.

### 3.6. Potential to Spread

Autumn fern is considered hardy in USDA zones 5–6, the cold temperate zone [25], enabling it to live more readily in the northeastern and central midwestern regions of the United States, where it is now being reported. This should allow it to naturalize farther north than where it now occurs in southern Ontario, Canada. There are three iNaturalist posts for the fern in southern Ontario: Ottawa on 6 June 2022 (observations/104501853); Point Edward on 29 July 2024 (observations/269363058); and Niagara Falls on 21 August 2024 (/observations/237371530). The autumn fern has also naturalized in Belgium and France [26], and there are iNaturalist posts for the Netherlands, Slovenia, and the United Kingdom (iNaturalist.com, accessed 10 May 2025).

### 3.7. Significance of This Naturalization

The significance of this naturalization of the autumn fern is unclear. Its ecological impacts are unknown, but the fern clearly displaces native vegetation where it is abundant (Figure 4D,E), and may influence the native biota (insects, etc.) associated with displaced native plants. In the more urban preserves and parks, the forest understories and creek banks where it grows have already been significantly altered by the presence of other non-native plants such as English ivy (*Hedera helix* L.), *Liriope* spp., and another common alien fern, *Macrothelypteris torresiana* (Gaudich.) Ching. Unfortunately, the history of intensive cotton cultivation in the Atlanta region and other parts of Georgia and the South appears to have caused the loss of the native herbaceous flora and their associated biota. But a comparison of properties in Pennsylvania with native tree canopies, but with and without native shrubs and native plant ground cover, found that those with native shrubs and native ground cover supported significantly more caterpillars and caterpillar species and significantly greater bird abundance, diversity, species richness, biomass, and breeding pairs of native species [27].

Except for Christmas fern, *Polystichum acrostichoides* (Michx.) Schott, and to a lesser degree, ebony spleenwort, *Asplenium platyneuron* (L.) Oakes ex D.C. Eaton, there are few native ferns growing on these rocky creeks and creek banks in the Atlanta, Georgia, region, the area with the most dense autumn fern populations. Perhaps these environments represent unoccupied niches for autumn fern [28]. For people who recognize and appreciate the difference between a natural environment without alien plants in nature preserves, the presence of autumn fern is significant, meaningful, and undesirable because it represents a degraded environment. In our opinion, the uniqueness and integrity of native environments are among the most defining and important qualities that places have. But for less knowledgeable visitors, the differences between natural environments dominated by autumn fern and those without it may not be apparent, but human awareness is probably not the most meaningful criterion.

### 3.8. Potential Actions for This Naturalization

Gillman [29] advocated that all species that have demonstrated the ability to self-reproduce, as autumn fern has, should be banned from sale and distribution. Although taking legal action to attempt to limit the further spread of the fern would be very desirable, it is probably not feasible in the United States. First of all, the dramatic spread of autumn fern in North America, as reported here, is recent and not well known. Even widely known invasive ferns such as Japanese climbing fern, *Lygodium japonicum* (Thunb. ex Murr.) Sw., which are Category 1 invasive plants (the most serious ranking) in both Georgia (https://gainvasivespeciescouncil.org/list/invasive-plants/, assessed 10 May 2025) and in Florida (https://www.floridainvasives.org/plant-list/2023-invasive-plant-species/, assessed 10 May 2025), can be freely purchased on the internet. A goal of this research is to alert and educate the scientific and conservation communities about this rapid increase and geographic spread of autumn fern. With time, the public will become better informed about the risks that this fern poses and will hopefully buy and plant it less. This, however, will be challenging due to autumn fern being the most marketed and planted, even though it may be among the most dangerous of temperate zone ferns.

The naturalization and invasion of ornamental ferns has been little studied or documented, except for research on the extremely invasive tropical *Salvinia molesta* D.Mitch. and *Lygodium micropyhllum* (Cav.) R Br. [30,31], and the efforts to document the occurrence of alien escaped ferns in Arkansas [13]. Recently, this has begun to change with research looking at the relationship between naturalization and market intensity [4,5], and the efforts to document the escape and spread of ornamental ferns in Georgia [6,7,32,33].

## 4. Materials and Methods

Two kinds of data were used to determine the distribution and abundance of the autumn fern in North America: herbarium collection records of wild-collected specimens and Research Grade iNaturalist posts of the fern (https://iNaturalist.com). iNaturalist is an online citizen science initiative of the California Academy of Sciences and the National Geographic Society, which began in 2008. iNaturalist members post their photographs with GPS locations and times of organisms that are supposed to be occurring naturally, as opposed to being in cultivation. The species in many of the better photographs are identified and designated as Research Grade by specialist curators and knowledgeable members. iNaturalist, with its massive number of observations (>250,000,000 at this writing), is valuable in biodiversity and invasive species research on species that are readily detectable and identifiable in photographs (https://iNaturalist.com) [12,33]. The primary source of herbarium collection records was the Southeast Regional Network of Expertise and Collections (SERNEC) (https://sernecportal.org, 10 May 2025), a consortium of 233 herbaria in the southeastern United States. While many of these herbaria focus on the Southeast, their collections are worldwide. In addition, herbarium collection data of the fern in the five largest herbaria in North America, including the US National Museum of Natural History Herbarium (US), the New York Botanical Garden (NY), the Missouri Botanical Garden (MO), Harvard University’s Grey Herbarium (GH), and the Field Museum of Natural History in Chicago (F) were evaluated on 10 May 2025. The iNaturalist posts of autumn fern used were from the first post in 2010 until the last date of data usage on 8 May 2025. The herbaria of SERNEC and the US National Museum had 76 specimens of *D. erythrosora* collected from North America. The only herbarium of the five largest herbaria in North America to hold collections of the fern from North America was the US National Museum of Natural History, which had four specimens of the fern. All four were duplicates of collections already accounted for, so a total of 76 herbarium localities and 2553 Research Grade iNaturalist posts were mapped. These collection records and iNaturalist posts were used to create a series of six maps to show the occurrence and distribution of the fern in different time periods, mostly at three-year intervals from 2008, shortly after it was discovered in Arkansas, to 1 October 2023. A seventh map was created to display the currently known distribution of autumn fern as of 16 May 2025.

To complement the descriptive analysis and to quantify the trend in observation rates, we applied a Poisson regression model to the iNaturalist post counts over five time periods (2012–mid-2025), using the number of months in each interval as an offset to account for unequal duration. The regression was performed using a generalized linear model (GLM) with a log link function (Poisson family), implemented in Python 3.10 using the statsmodels package (version 0.14.0, released October 2023).

## 5. Conclusions

This study documents the remarkably rapid spread and great increase in the autumn fern in North America for the first time. These novel findings will promote research to continue to monitor the autumn fern’s expansion and determine its potential ecological impacts. These findings should also help alert and educate the public about the potential risks of continuing to buy and plant what is currently the most marketed and perhaps most dangerous invasive temperate fern.

## Data Availability

The data used from the mapping are herbarium specimens of *Dryopteris erythrosora* accessible through the SERNEC portal (https://sernecportal.org/portal/collections/index.php, accessed on 10 May 2025). The iNaturalist posts used for the mapping can be retrieved by searching *Dryopteris erythrosora* in the United States and Canada with an end date of 10 May 2025 (https://iNaturalist.com).
